# Genetic diversity, population structure, and phylogenetic relationships of a widespread East Asia herb, *Cryptotaenia japonica* Hassk. (Apiaceae) based on genomic SNP data generated by dd-RAD sequencing

**DOI:** 10.3389/fgene.2024.1368760

**Published:** 2024-08-14

**Authors:** Baocheng Wu, Jun Wen, Ruisen Lu, Wei Zhou

**Affiliations:** Jiangsu Key Laboratory for the Research and Utilization of Plant Resources, Institute of Botany, Jiangsu Province and Chinese Academy of Sciences (Nanjing Botanical Garden Mem. Sun Yat-Sen), Nanjing, Jiangsu, China

**Keywords:** RAD-seq, *Cryptotaenia japonica*, Apiaceae, genetic diversity, population structure, gene flow, SNP, dd-RAD sequencing

## Abstract

Single-nucleotide polymorphisms (SNPs) represent the most prevalent form of genomic polymorphism and are extensively used in population genetics research. Using dd-RAD sequencing, a high-throughput sequencing method, we investigated the genome-level diversity, population structure, and phylogenetic relationships among three morphological forms of the widely distributed taxon *Cryptotaenia japonica* Hassk., which is native to East Asia. Our study aimed to assess the species status of *C. japonica* according to its genetic structure and genetic diversity patterns among 66 naturally distributed populations, comprising 26 *C. japonica* f. *japonica*, 36 *C. japonica* f. *dissecta* (Y. Yabe) Hara and 4 *C. japonica* f. *pinnatisecta* S. L. Liou accessions. Based on genomic SNP data generated by dd-RAD sequencing, we conducted genetic diversity, principal component, neighbor-joining (NJ) phylogenetic, admixture clustering, and population differentiation analyses. The findings revealed the following: (1) 5,39,946 unlinked, high-quality SNPs, with mean *π*, *H*_O,_ *H*_E_ and *F*_IS_ values of 0.062, 0.066, 0.043 and −0.014, respectively, were generated; (2) population divergence was unaffected by isolation through distance; (3) six main distinct regions corresponding to geographic locations and exhibiting various levels of genetic diversity were identified; (4) pairwise *F*_ST_ analysis showed significant (*P* < 0.05) population differentiation in 0%–14% of populations among the six regions after sequential Bonferroni correction; and (5) three migration events (historical gene flow) indicated east‒west directionality. Moreover, contemporary gene flow analysis using Jost’s D, Nei’s *G*_ST_, and *Nm* values highlighted the middle latitude area of East Asia as a significant contributor to genetic structuring in *C. japonica*. Overall, our study elucidates the relatively low genetic differentiation and population structure of *C. japonica* across East Asia, further enhancing our understanding of plant lineage diversification in the Sino-Japanese Floristic Region.

## 1 Introduction

Genetic diversity is one of the important pillars of biodiversity, and high genetic diversity increases wild plants’ ability to survive and reduces the risk of extinction for species and allows the prediction of species fitness based on the study of genetic diversity (Phillips et al., 2012; Hu et al., 2022). In addition, genetic differentiation and gene flow are important elements in understanding the evolutionary and adaptive potential of populations, while high gene flow reduces the incidence of inbreeding and population differentiation by increasing the exchange of genetic material between populations (Waqar et al., 2021). Plant genetic diversity is influenced by seed dispersal, reproductive systems, life history, geographic range and evolutionary history.

Previous studies have focused mainly on genetic diversity, population structure, and genetic characterization based on chloroplast DNA sequences, single-copy nuclear genes and microsatellite markers. Over the past few decades, researchers have increasingly used next-generation sequencing, which is becoming more affordable, and markers based on sequencing can be assessed throughout the genome (e.g., using RAD sequencing). SNPs from double-digest restriction site-associated DNA sequencing (ddRAD-seq) are low-mutation-rate markers. An increasing number of studies have used next-generation sequencing (NGS) and genotyping-by-sequencing (GBS) to investigate single nucleotide polymorphisms (SNPs), which have become markers of choice because they are abundant and stable in the genome. GBS is based on genome-wide sequencing of loci adjacent to conservative restriction sites, which makes it possible to obtain thousands of homologous loci for species with no prior genomic data (Shekhovtsov et al., 2022). It has certain applications in populations of *Capparis spinosa* L. Wang and Zhang (2022), *Trigonobalanus doichangensis* (A. Camus) Forman (Hu et al., 2022), *Hedyotis chrysotricha* (Palib.) Merr. Yuan et al. (2022), *Prunus* L. Li et al. (2020), *Epimedium* Tourn. ex L. Zhang et al. (2023), *Cycas* L. He et al. (2023), *Camellia granthamiana* Sealy (Chen et al., 2023), Saxifraga Tourn. ex L. Yuan et al. (2023), *Pterocarya hupehensis* Skan (Lu et al., 2024), *Fitzroya* Lindl. Cano et al. (2022), *Hopea reticulata* Tardieu (Tang et al., 2024), *Soldanella* L. Slovák et al. (2022), *Bruguiera gymnorhiza* (L.) Lam. ex Savigny Ruang-areerate et al. (2023), *Pisum sativum* L. Rispail et al. (2023) and other plants.

The Sino-Japanese Floristic Region of East Asia harbors the most diverse temperate flora in the world and was the most important glacial refuge for Tertiary representatives (‘relics’) throughout Quaternary ice-age cycles (Qiu et al., 2011). *Cryptotaenia* DC (Apiaceae) is a polyphyletic genus with three species in the tribe Pimpinelleae and four in the tribe Oenantheae (Spalik and Downie, 2007). *Cryptotaenia japonica* Hassk. in the tribe Oenantheae is present in regions that are important glacial refuges: *C. japonica* is endemic to East Asia (China, Japan and the Korean Peninsula) (Spalik and Downie, 2007). In Japan, *C. japonica* is known as “Mitsuba” and is used as a condiment (seeds) or a garnish (tender leaves) (Okuno et al., 2017), and in China, it is known as “Ya-er-qin” and is used as a tonic to strengthen the human body and as a vegetable (WU et al., 2014). It is treated as a species with three similar forms, *C. japonica* f. *japonica*, *C. japonica* f. *dissecta* (Y. Yabe) Hara, and *C. japonica* f. *pinnatisecta* S. L. Liou, because it is a distinctive, widespread taxon exhibiting almost continuous variation in leaves and inflorescences across its range (Pan and Watson, 2005).

In the present study, we used SNP markers from dd-RAD sequencing, admixture clustering, principal component analysis (PCA), neighbor-joining (NJ) phylogenetic analysis and gene flow methods to comprehensively investigate the mechanisms underlying the genetic diversity and distribution differences of *C. japonica* across a large spatial scale in East Asia. Thus, we aimed to (1) elucidate the population structure and intraspecific divergence of the lineages of *C. japonica* Hassk. (2) To adequately reveal the spatial pattern of genetic diversity among identified geographical regions, and (3) to provide a reference for future research on the diversity of widespread plants in Asia.

## 2 Materials and methods

### 2.1 Taxon sampling and identification of samples

**FIGURE 1:**
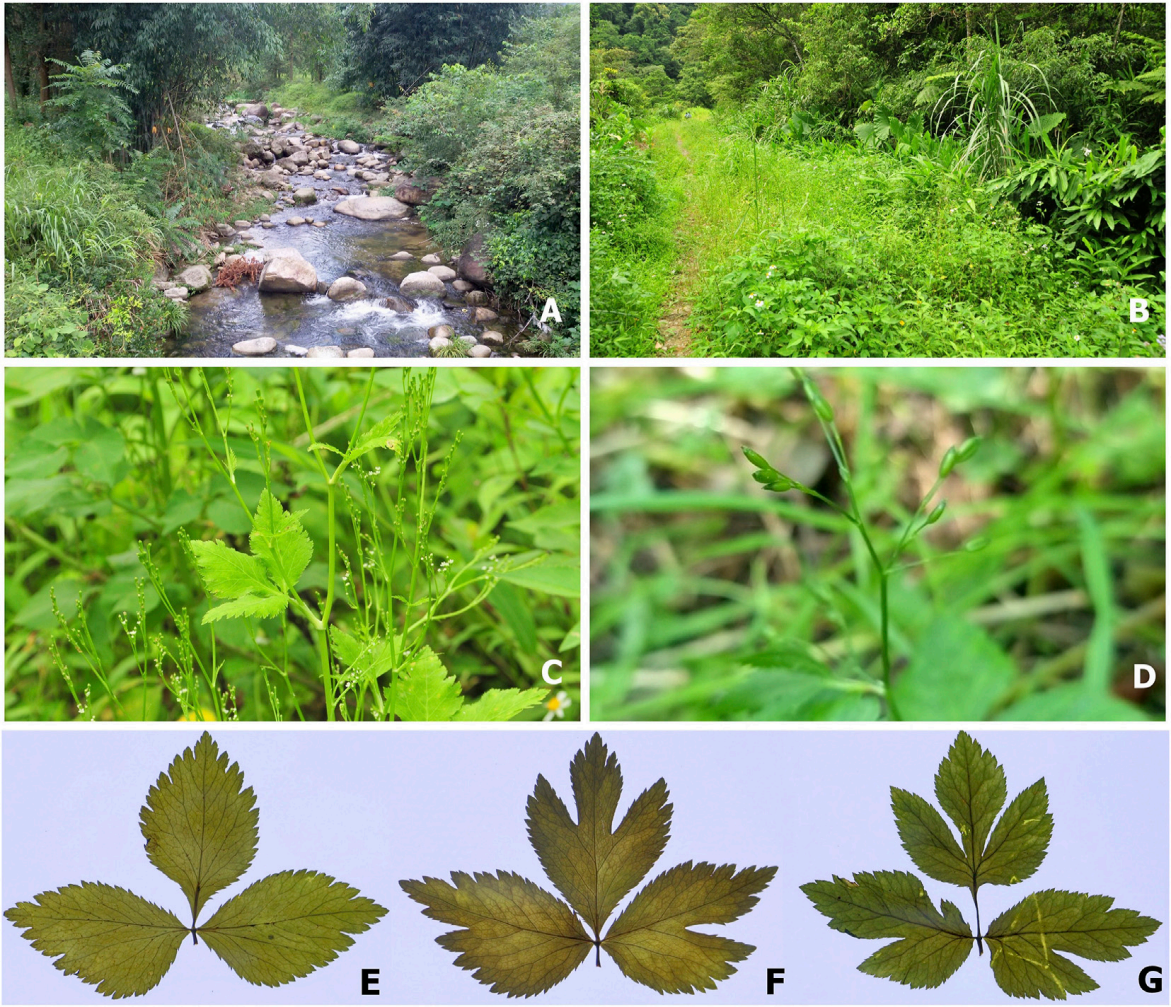
*Cryptotaenia japonica* Hassk. Different habitats (A: damp places along ditches or streams, **(B)** damp places in forests, along the trail). Adult plant with flowers and young fruits **(C)**. Fruits **(D)**. The leaves of three forms: *C. japonica* f*. japonica* **(E)**, *C. japonica* f. *dissecta* (Y. Yabe) Hara **(F)**, and *C. japonica* f. *pinnatisecta* S. L. Liou **(G)**.

**FIGURE 2:**
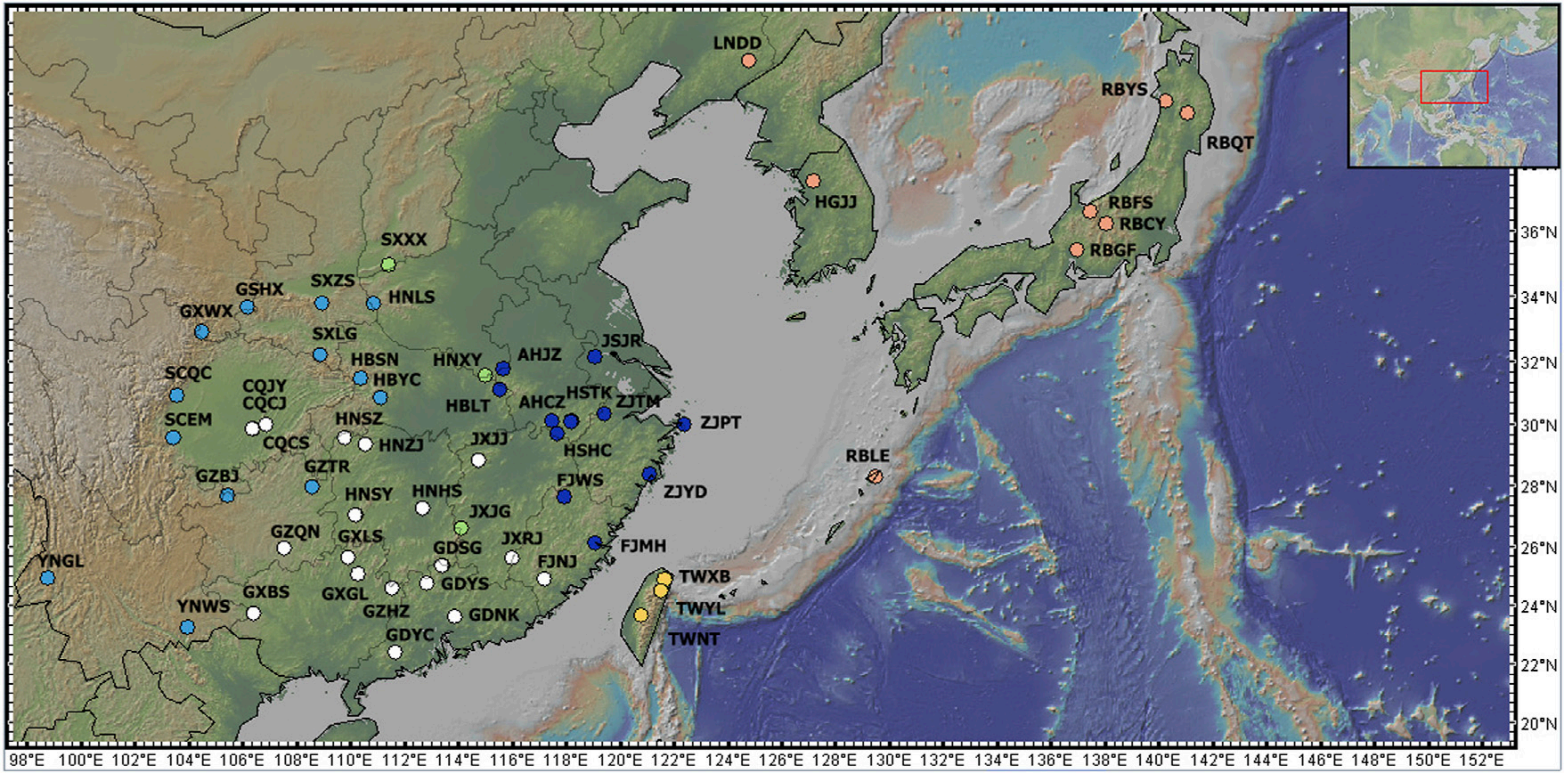
Sampling points for *C. japonica* populations. The pink dots show the populations in the NA region, the yellow dots indicate the TW region, the dark blue dots indicate the EC region, the green dots indicate the CC region, the sky blue dots indicate the WC region, and the white dots indicate the SC region. The map was produced by GeoMapApp (http://www.geomapapp.org/).

**FIGURE 3:**
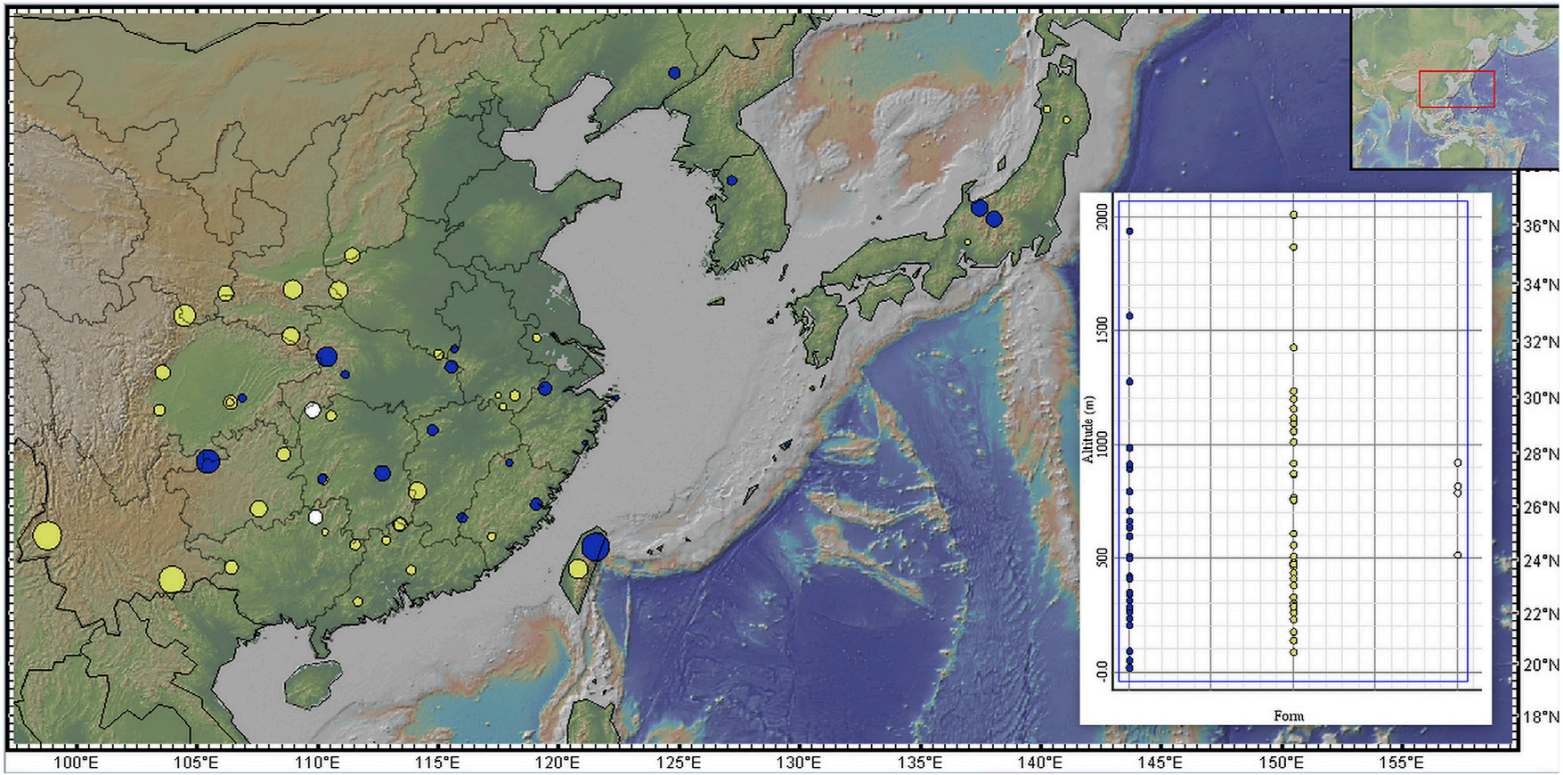
Distribution of forms across elevations. Blue dot = *C. japonica* f. *japonica*; yellow dot = *C. japonica* f. *dissecta*; white dot = *C. japonica* f. *pinnatisecta*. The size of the dot is larger, and the altitude is greater on the map. The map was produced by GeoMapApp (http://www.geomapapp.org/).

South, West and East China, the Korean Peninsula and Japan were considered the three major distribution regions of *C. japonica* Hassk. Fresh leaves, especially young leaves, were collected from 1-6 individuals at each location and desiccated in silica gel. The dry leaves were stored at −20°C until use. Vouchers were deposited in the Herbarium of the Institute of Botany, Jiangsu Province and the Chinese Academy of Sciences (NAS). We identified the samples as subspecies using previously described methods (Shan and Sheh, 1985; Liou, 1990). In this study, a total of 179 wild individuals representing 26 populations of *C. japonica* f. *japonica*, 36 populations of *C. japonica* f. *dissecta* (Y. Yabe) Hara, 4 populations of *C. japonica* f. *pinnatisecta* S. L. Liou and 1 population of *C. canadensis* were collected (Figure 1). The 176 samples we studied were collected from 66 locations in 3 countries across the entire natural range of this species, including Mainland China, Taiwan Island, Korean Peninsula and Japan Islands. Our samples of *C. japonica* included all the proposed forms. In addition, 3 individuals of *C. canadensis* (L.) DC. from 1 wild population were used in the study as an outgroup (Table 1; Figures 2, 3).

**TABLE 1:** Localities of the three different forms of *C. japonica* and *C. canadensis* populations sampled.

Region	Population name	Forms	Population code	N	Locality	Longitude (E °)	Latitude (N °)	Altitude (m)
EC	AHJZ	*C. japonica* f. japonica	6	3	Jinzhai, Anhui, China	115.68	31.75	231
	HBLT	*C. japonica* f. japonica	44	3	Dabieshan, Hubei, China	115.55	31.11	659
	AHCZ	*C. japonica* f. dissecta	5	4	Shitai, Anhui, China	117.49	30.10	135
	FJMH	*C. japonica* f. japonica	55	3	Minhouxian, Fujian, China	119.07	26.11	593
	FJWS	*C. japonica* f. japonica	56	3	Wuyishan, Fujian, China	117.95	27.65	203
	HSHC	*C. japonica* f. dissecta	4	3	Qimen, Anhui, China	117.67	29.69	227
	HSTK	*C. japonica* f. japonica	2	3	Huangshan, Anhui, China	118.22	30.10	311
		*C. japonica* f. dissecta	1	3	Huangshan, Anhui, China	118.20	30.09	326
		*C. japonica* f. dissecta	3	3	Huangshan, Anhui, China	118.18	30.08	462
	JSJR	*C. japonica* f. dissecta	60	2	Baohuashan, Jiangsu, China	119.08	32.13	284
	ZJPT	*C. japonica* f. japonica	59	2	Putuoshan, Zhejiang, China	122.39	30.01	48
	ZJTM	*C. japonica* f. japonica	58	3	Tianmushan, Zhejiang, China	119.43	30.34	704
	ZJYD	*C. japonica* f. japonica	57	2	Yandangshan, Zhejiang, China	121.10	28.37	88
WC	GSHX	*C. japonica* f. dissecta	13	3	Huixian, Shaanxi, China	106.19	33.68	913
	GSWX	*C. japonica* f. dissecta	14	3	Wenxian, Shaanxi, China	104.48	32.91	1,422
	GZBJ	*C. japonica* f. japonica	62	3	Bijieshi, Guizhou, China	105.44	27.69	1,561
	GZTR	*C. japonica* f. dissecta	20	3	Fanjingshan, Guizhou, China	108.59	27.96	749
	HBSN	*C. japonica* f. japonica	43	3	Shenlongjia, Hubei, China	110.39	31.47	1,272
	HBYC	*C. japonica* f. japonica	42	3	Wushan, Hubei, China	111.14	30.84	260
	HNLS	*C. japonica* f. dissecta	7	3	Lushi, Henan, China	110.86	33.78	1,153
	SCEM	*C. japonica* f. dissecta	16	2	Emeishan, Sichuan, China	103.44	29.57	554
	SCQC	*C. japonica* f. dissecta	15	2	Qingchengshan, Sichuan, China	103.57	30.91	868
	SXLG	*C. japonica* f. dissecta	11	2	Langao, Shaanxi, China	108.88	32.20	1,090
		*C. japonica* f. dissecta	12	2	Langao, Shaanxi, China	108.89	32.19	1,054
	SXZS	*C. japonica* f. dissecta	10	3	Zhashui, Shaanxi, China	108.97	33.82	1,195
	YNGL	*C. japonica* f. dissecta	61	3	Gaoligongshan, Yunnan, China	98.78	24.93	2005
	YNWS	*C. japonica* f. dissecta	22	3	Laojunshan, Yunnan, China	103.96	23.27	1863
CC	HNXY	*C. japonica* f. dissecta	8	3	Xinxian, Henan, China	115.02	31.55	431
	JXJG	*C. japonica* f. dissecta	46	6	Jinggangshan, Jiangxi, China	114.13	26.60	1,113
	SXXX	*C. japonica* f. dissecta	9	3	Xiaxian, Shanxi, China	111.43	34.98	864
TW	TWNT	*C. japonica* f. dissecta	66	3	Nantouxian, Taiwan, China	120.80	23.67	1,230
	TWXB	*C. japonica* f. japonica	64	3	Xinbeishi, Taiwan, China	121.88	25.02	15
	TWYL	*C. japonica* f. japonica	68	3	Taipingshan, Taiwan, China	121.53	24.50	1933
NA	HGJJ	*C. japonica* f. japonica	73	3	Gyeonggi-do, Gwangiu-si, Korea	127.18	37.48	407
	LNDD	*C. japonica* f. japonica	74	4	Kuandianxian, Liaoning, China	124.80	40.94	563
	RBCY	*C. japonica* f. japonica	79	3	Nagano, Chubu, Japan	138.06	36.21	909
	RBFS	*C. japonica* f. japonica	82	2	Toyama, Chubu, Japan	137.46	36.58	981
	RBGF	*C. japonica* f. dissecta	83	4	Sakahogi-cho, Gifu, Japan	136.98	35.44	84
	RBLE	*C. japonica* f. japonica	75	3	Amami, Kagoshima, Japan	129.48	28.30	15
	RBQT	*C. japonica* f. dissecta	78	3	Akita, Touhoku, Japan	140.26	39.80	226
	RBYS	*C. japonica* f. dissecta	77	3	Iwate, Touhoku, Japan	141.06	39.46	181
SC	CQCJ	*C. japonica* f. dissecta	18	2	Jinyunshan, Chongqing, China	106.36	29.84	258
	CQJY	*C. japonica* f. japonica	17	3	Jinyunshan, Chongqing, China	106.39	29.84	763
	CQCS	*C. japonica* f. japonica	71	2	Changshou, Chongqing, China	106.88	30.00	282
	FJNJ	*C. japonica* f. dissecta	54	3	Nanjingxian, Fujian, China	117.22	24.91	287
	GDNK	*C. japonica* f. dissecta	51	3	Nankunshan, Guangdong, China	113.88	23.64	406
	GDSG	*C. japonica* f. japonica	48	1	Lechagnshi, Guangdong, China	113.42	25.37	632
		*C. japonica* f. pinnatisecta	49	1	Lechagnshi, Guangdong, China	113.42	25.36	783
		*C. japonica* f. dissecta	50	2	Lechagnshi, Guangdong, China	113.41	25.36	605
	GDYC	*C. japonica* f. dissecta	52	2	Baiyong, Guangdong, China	111.67	22.42	376
	GXBS	*C. japonica* f. dissecta	37	2	Dawangling, Guangxi, China	106.41	23.74	754
	GXGL	*C. japonica* f. dissecta	31	2	Guilin, Guangxi, China	110.3	25.08	172
	GXHZ	*C. japonica* f. japonica	35	2	Guposhan, Guangxi, China	111.56	24.60	505
		*C. japonica* f. dissecta	36	3	Guposhan, Guangxi, China	111.56	24.60	504
		*C. japonica* f. pinnatisecta	34	2	Guposhan, Guangxi, China	111.56	24.60	510
	GXLS	*C. japonica* f. japonica	32	1	Longsheng, Guangxi, China	109.91	25.63	790
		*C. japonica* f. pinnatisecta	33	2	Longsheng, Guangxi, China	109.91	25.62	812
	GDYS	*C. japonica* f. dissecta	53	3	Yangshan, Guangdong, China	112.84	24.76	298
	GZQN	*C. japonica* f. dissecta	21	2	Dushanxian, Guizhou, China	107.55	25.94	1,006
	HNHS	*C. japonica* f. japonica	41	2	Hengshan, Hunan, China	112.68	27.26	890
	HNSY	*C. japonica* f. japonica	39	2	Dongkouxian, Hunan, China	110.20	27.05	338
		*C. japonica* f. dissecta	38	3	Dongkouxian, Hunan, China	110.22	27.04	477
	HNSZ	*C. japonica* f. pinnatisecta	69	3	Sangzhixian, Hunan, China	109.79	29.57	916
	HNZJ	*C. japonica* f. dissecta	40	2	Zhangjiajie, Hunan, China	110.56	29.36	470
	JXJJ	*C. japonica* f. japonica	45	3	Xiushuixian, Jiangxi, China	114.77	28.84	495
	JXRJ	*C. japonica* f. japonica	47	2	Ruijinshi, Jiangxi, China	115.99	25.61	416
OUTG		*C.* canadensis	23	3	Newark, Ohio, United States			

### 2.2 DNA extraction and quality control

Total genomic DNA was isolated from leaves using a TIANGEN DP305 DNA extraction kit. Extracted DNA was purified with Qiagen DNA Purification Kits (Oiagen, Inc., Valencia, CA, United States). The concentration and quality of the purified DNA samples were checked by a OneDrop™ *OD-1000 +* spectrophotometer (www.onedrop.cn) and 1% agarose gel electrophoresis. Finally, 179 of the 212 collected DNA samples met the minimum quality requirements in this study and were ready for subsequent sequencing and genotyping.

### 2.3 Double digest restriction site associated DNA sequencing (ddRAD-seq) library preparation and sequencing

Only when the quality of the total genomic DNA was >100 ng/μL could it be used for the ddRAD-seq library. The libraries of each individual were sequenced by Genepioneer Biotechnologies Co., Ltd. (Nanjing, China) via an Illumina NovaSeq 6,000 System sequencer and VAHTS Universal DNA Library Prep Kit for Illumina (ND607, bio.vazyme.com). The enzymes EcoRI (Read1, G^AATTC) and NlaIII (Read2, Hin1II, CATG^) were used for DNA digestion, and the fragments were ligated to a barcode adaptor and a common adaptor with compatible sticky ends. The target fragments were kept within the range of 300–500 bp in size. Then, 150 bp paired-end reads were generated, and approximately 216 GB raw data of 179 individuals was generated.

A quality control pipeline was used to process the raw data by FASTP software (version: 0.20.0), and its own script was used to filter the raw data to obtain clean data. The parameter was set to q 5 -n 5 (Chen et al., 2018). Then, the function TRIMFQ in SEQTK software (version: 1.3-r106) was used to obtain high-quality data (https://github.com/lh3/seqtk.git).

The software pipeline STACKS (Catchen et al., 2011; Catchen et al., 2013; Paris et al., 2017; Rochette and Catchen, 2017; Rochette et al., 2019) was used to process the filtered high-quality data from the ddRAD data. We genotyped and once again identified the loci from short-read sequences by using the STACKS 2.59 pipeline. Briefly, all sequences were processed by USTACKS (--deleverage -m = 3 and -M = 4 - p 10 - t gzfastq -d–R) for RAD tags, CSTACKS (-n 4 -p 40) was used to construct a catalog of consensus loci including all the loci from *D. japonica* samples and merge all alleles together, SSTACK was used to compare against the catalog with sets of stacks that were created in USTACKS, TSV2BAM was used to transfer tsv files to BAM files, we aligned the reads to the locus and called SNPs by GSTACKS, and POPULATIONS (populations -P stacks--popmap popmap.list -p 2 -t 25 --write-random-snp--vcf--fasta-samples--fasta-loci) was used to filter the catalog of reads to produce a dataset for subsequent analyses. After STACKS processing, we obtained 4,504,050 SNPs. VCFtools software (version: 0.1.16) (Danecek et al., 2011) was used to filter the loci based on 1) --min-alleles 2, --max-alleles 2; 2) --max-missing 0.50; 3) --mac3; and 4) --minDP 3. Finally, a total of 539,946 SNPs were obtained after VCFTOOLS processing.

### 2.4 Population genetic diversity, geographical patterns, region structure, and admixture

We calculated the nucleotide diversity (*π*), observed heterozygosity (*H*_O_), expected heterozygosity (*H*_E_), and inbreeding coefficient (*F*_IS_) for each population using the POPULATION program in STACKS (version: 5.39). We calculated pairwise *F*-statistics (*F*_ST_) (1,000 permutations) among populations by using ARLEQUIN (version: 3.5.2.2) with sequential Bonferroni correction (Excoffier et al., 2005). *F*_ST_ was used as a measure of genetic distances among pairs of populations using the POPULATION program in STACKS (version: 5.39), and the transformation *F*_ST_/(1-*F*_ST_) was applied. Geographical distances included pairwise shortest distances among populations by using the geosphere program (version: 1.5–14) (https://github.com/phiala/ecodist.git) in the R package (version: 3.6.2). Mantel tests were used to measure the correlation between genetic and geographical distances among populations. Mantel tests were performed with the ecodist program (version: 2.0.7) in the R package (version: 3.6.2) (Goslee and Urban, 2007; Goslee, 2009). Graphics were created by the ggplot2 program (version 3.3.3) in the R package (version: 3.6.2) (https://github.com/tidyverse/ggplot2.git).

Three methods were used to infer overall patterns of population genetic structure. Firstly, all the data were used for phylogenetic inference by MEGA (version: 11.0.7) (Tamura et al., 2021) to obtain phylogenetic trees based on the NJ method. Secondly, we used GCTA software (Genome-wide Complex Trait Analysis, version: 1.93.2) (Yang et al., 2011) for PCA based on the function (--make-grm--autosome). The GRM of GCTA was used to estimate genetic relationships among individuals from SNP data. Finally, we used ADMIXTURE (version: 1.3.0) (Alexander et al., 2009), which implements a model-based approach to infer populations and individuals in a maximum-likelihood (ML) framework. ADMIXTURE outperforms several other software programs in terms of analysis efficiency for genome-wide SNP data, which are sometimes large. We estimated ancestry coefficients for every individual in 10 replicate software runs for each of *K* = 1–20. Then, we estimated two replicate runs of 50-fold cross-validation for *K* = 1–20 to determine the potential error in each *K*. The *K* value with the lowest cross-validation error was the best *K*. Each individual in this study was assigned to a cluster for the best *K* model.

### 2.5 Gene flow analysis

Three methods were used to analyze the gene flow between different regions of *C. japonica* in East Asia. Firstly, TREEMIX (version: 1.13) (Pickrell and Pritchard, 2012) was used to infer splitting and mixture patterns among populations of *C. japonica*. Migration events (m) from 0–12 were specified between populations, and 10 iterations per m were tested. In this analysis, the “-bootstrap 1,000”, “-m 0–12” and “-root” parameters were used to construct an ML tree. Secondly, we ran BA3-SNPs-autotune (version: 2.1.2) in BAYESASS3-SNPs (version: 1.1) (Wilson and Rannala, 2003; Mussmann et al., 2019) with the default delta values for allelic frequency, migration rates, and inbreeding coefficients by using the data of six regions, with *C. canadensis* (L.) DC. as the outgroup, based on -r 10-g 10,000-b 1,000 parameters. Thirdly, diveRsity software (version: 1.9.90) (Keenan et al., 2013) of the R package (version: 3.6.2) was also used to analyze the relative direction of the gene flow between these six regions by calculating Jost’s D, Nei’s *G*_ST_, and *Nm*. The 95% confidence intervals were calculated from 1,000 bootstrap replicates to test for asymmetric flow (significantly higher in one direction than in the other).

## 3 Results

### 3.1 RAD-seq data and SNP filtering

Approximately 742 million (741, 584, 140) raw reads were produced from 179 sampled individuals of *Cryptotaenia*. After quality control, filtering, and trimming, 679.72 million high-quality reads were retained. A catalog containing 494, 675, 435 loci was constructed, and 28, 89, 851 loci were genotyped by GSTACKS using the datasets of all *Cryptotaenia* samples. The mean, minimum, and maximum values for effective per-sample coverage were 34.7×, 15.2×, and 96.1×, respectively. After filtering low-quality loci (minor allele frequency < 0.01; missing rate > 0.5), 5,39,946 unlinked SNPs were eventually identified and used for all subsequent analyses.

### 3.2 Population variation

Using genome-wide SNP markers, we detected some levels of genetic diversity in *C. japonica* from East Asia. The values of *π* ranged from 0.016 to 0.120, and the mean was 0.062; the values of *H*_O_ ranged from 0.020 to 0.158, and the mean was 0.066; the values of *H*_E_ ranged from 0.012 to 0.094, and the mean was 0.043; and the values of *F*_IS_ ranged from −0.095 to 0.051, and the mean was −0.014, including *F*_IS_ values of 42 populations that were negative (Table 2).

**TABLE 2:** Genetic characteristics of the sampled populations.

Region	Population	*π*	*H*O	*H*E	*F*IS	Region	Population	*π*	*H*O	*H*E	*F*IS
EC	AHJZ	0.070	0.086	0.054	−0.027	TW	TWYL	0.052	0.024	0.041	0.051
	HBLT	0.085	0.088	0.066	−0.007	NA	HGJJ	0.033	0.029	0.025	0.007
	AHCZ	0.065	0.060	0.053	0.007		LNDD	0.018	0.024	0.014	−0.011
	FJMH	0.100	0.157	0.079	−0.094		RBCY	0.045	0.031	0.035	0.026
	FJWS	0.111	0.155	0.087	−0.071		RBFS	0.017	0.021	0.012	−0.006
	HSHC	0.018	0.025	0.014	−0.012		RBGF	0.016	0.023	0.013	−0.011
	HSTK	0.079	0.080	0.073	−0.002		RBLE	0.043	0.027	0.033	0.029
	JSJR	0.068	0.069	0.047	−0.001		RBQT	0.045	0.022	0.035	0.042
	ZJPT	0.072	0.073	0.050	−0.002		RBYS	0.017	0.022	0.012	−0.009
	ZJTM	0.087	0.089	0.065	−0.005	SC	CQCJ	0.080	0.111	0.057	−0.048
	ZJYD	0.075	0.074	0.050	0.000		CQJY	0.087	0.116	0.061	−0.044
WC	GSHX	0.075	0.090	0.058	−0.027		CQCS	0.029	0.023	0.023	0.012
	GSWX	0.081	0.087	0.063	−0.01		FJNJ	0.101	0.156	0.080	−0.091
	GZBJ	0.029	0.030	0.022	−0.001		GDNK	0.101	0.158	0.080	−0.095
	GZTR	0.075	0.117	0.060	−0.07		GDSG	0.093	0.117	0.077	−0.038
	HBSN	0.070	0.055	0.055	0.028		GDYC	0.110	0.157	0.079	−0.071
	HBYC	0.073	0.088	0.057	−0.027		GXBS	0.024	0.026	0.016	−0.002
	HNLS	0.036	0.024	0.028	0.022		GXGL	0.082	0.116	0.059	−0.051
	SCEM	0.069	0.071	0.048	−0.002		GXHZ	0.047	0.058	0.042	−0.023
	SCQC	0.077	0.072	0.054	0.008		GXLS	0.084	0.111	0.067	−0.043
	SXLG	0.087	0.093	0.072	−0.011		GDYS	0.120	0.156	0.094	−0.057
	SXZS	0.051	0.056	0.040	−0.008		GZQN	0.086	0.121	0.061	−0.053
	YNGL	0.017	0.023	0.013	−0.010		HNHS	0.027	0.024	0.019	0.005
	YNWS	0.018	0.024	0.013	−0.011		HNSY	0.044	0.031	0.038	0.031
CC	HNXY	0.019	0.026	0.014	−0.012		HNSZ	0.019	0.025	0.014	−0.008
	JXJG	0.017	0.022	0.014	−0.009		HNZJ	0.022	0.026	0.015	−0.006
	SXXX	0.018	0.024	0.014	−0.010		JXJJ	0.050	0.055	0.039	−0.008
TW	TWNT	0.045	0.031	0.036	0.026		JXRJ	0.022	0.021	0.014	0.002
	TWXB	0.018	0.026	0.014	−0.012	OUTG		0.100	0.149	0.079	−0.081

Abbreviations: *π*, nucleotide diversity; *H*_O_, observed heterozygosity; *H*_E_, expected heterozygosity; *F*_IS_, within-population inbreeding coefficient.

### 3.3 AMOVA and mantel test results

For the 57 natural geographical populations of *C. japonica*, analysis of molecular variance (AMOVA) revealed that most (55.55%) of the observed genetic variation was among the populations (Table 3). However, 44.45% of the total genetic diversity was attributable to within-individual local population variation (*p* < 0.01), consistent with the significant genetic differentiation among these 57 populations.

**TABLE 3:** AMOVA of genetic variation among 57 populations of *C. japonica*.

Source of variation	d. f	Sum of squares	Variance components	Percentage of Variation (%)
Among populations	56	211,664.061	543.17255	55.55
Within populations	295	128,238.607	434.70714	44.45
Total	351	339,902.668	977.87969	100

Note: *p* < 0.01.

**FIGURE 4:**
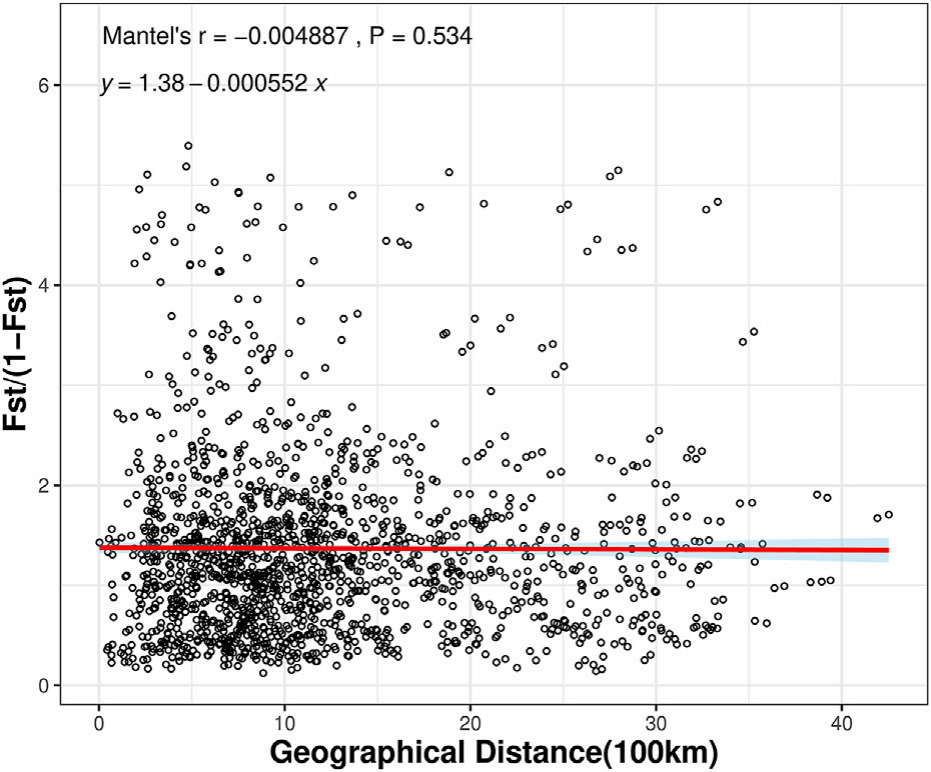
Mantel tests between genetic distance and geographic distance among the *C. japonica* populations.

To investigate whether geographic distance contributed to the observed genetic differentiation among the 57 populations, we determined the relationship between geographic distance and genetic distance for all pairs of populations analyzed here using the Mantel test method. The results indicated that there was no significant correlation between genetic distance and geographic distance among the 57 populations (r = −0.004887, *P* = 0.534) (Figure 4). The results suggested that spatial distance was not an important factor shaping the genetic structure among wild populations of *C. japonica*. No isolation by distance (IBD) pattern was found in 57 populations (Figure 4).

### 3.4 Genetic structure and geographical patterns

**FIGURE 5:**
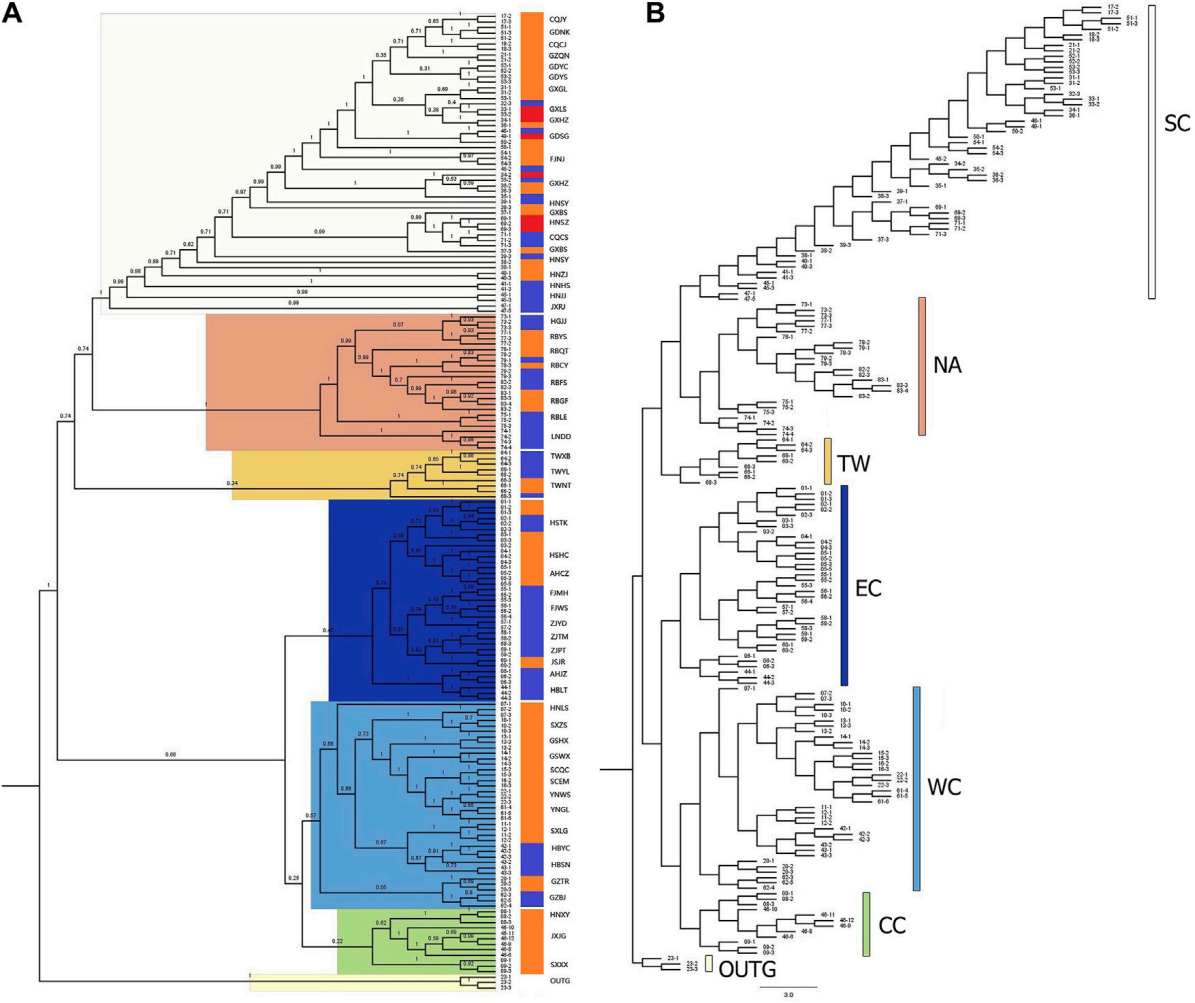
Neighbor-joining (NJ) phylogenetic tree of 176 *C. japonica* plants with *C. canadensis* as the OUTG. **(A)** Consensus NJ-tree with bootstrap values, **(B)** NJ-tree with branch length. The three color gradient bars in **(A)** show 3 forms of *C. japonica* for each individual, where blue indicates *C. japonica* f. *japonica*, orange indicates *C. japonica* f. *dissecta*, and red indicates *C. japonica* f. *pinnatisecta*. The color scheme in **(B)** also corresponds to the highlights of each branch in **(A)** and the dot color in Figure 2.

To further determine the phylogenetic relationships among these populations, we constructed NJ trees based on SNPs. The NJ trees revealed polyphyly in *C. japonica,* which could be separated into two clades with moderate posterior support (Figures 5A, B). *C. canadensis,* representing the outgroup (OUTG) lineage, diverged first from other lineages. Geographically, there was a propensity for serial divergence in *C. japonica* from southern to northern East Asia. One clade included the SC (South China), NA (Northeast Asia), and TW (Taiwan islands) populations, and the other clade included the EC (East China), WC (West China), and CC (Central China) populations (Figures 2, 5).

The SC region includes most of southern China (part of Fujian, Guangdong, and Guangxi Provinces) and part of Jiangxi, Hunan and Chongqing Provinces of China, with 19 populations (bootstrap support 100%). The NA region includes part of Northeast China, the Korean Peninsula, and the Japanese Islands, with 8 populations (bootstrap support 100%). The TW region includes the Taiwan Province of China, which has 3 populations (with 34% bootstrap support). The EC region includes parts of Hubei, Anhui, Jiangsu, Zhejiang, and northern Fujian Provinces in China, with 11 populations (47% bootstrap support). The WC region includes part of the Henan, Hubei, Guizhou, Gansu, Shaanxi, Sichuan, and Yunnan Provinces of China, with 13 populations (bootstrap support 57%). Specifically, the CC region includes parts of Jiangxi, Henan and Shanxi Provinces of China, with 3 populations (bootstrap support 22%), where the JXJG population is located in southern Jiangxi Province. The individuals of *C. japonica* in the SC region had longer branches than the other individuals in the other five regions (Figure 5B).

All the individuals contained in the population are grouped together with their corresponding populations in the CC, WC, and EC regions. However, we also found that individuals 68–3 (TWYL) in the TW region and 53–1 (GDYS), 39–1, 38–3 (HNSY) and 45–2 (JXJJ) in the SC region did not cluster with other individuals in the population. The individuals from the RBCY (79) and RBQT (78) populations are admixed in the NA region branch. In the phylogenetic tree, we still found that the above individuals were still near the respective populations in their regions (Figures 5A, B).

**FIGURE 6:**
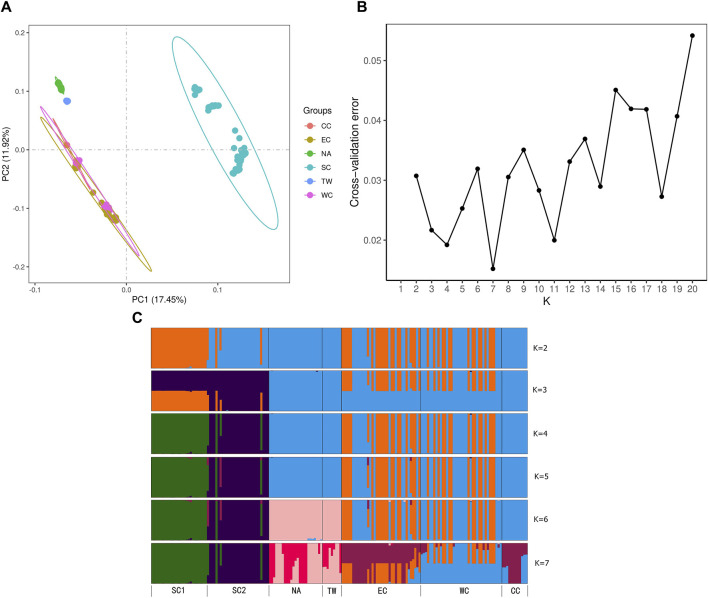
**(A)** Principal component analysis (PCA) displaying the first two axes (PC1 and PC2). **(B)** Values of the cross-entropy criterion for numerous clusters ranging from *K* = 1 to 20. **(C)** Bar plot of ancestry coefficients for *K* = 2 to 7. The dtailed information of six regions could be found in Table 1, 2 and Figures 2.

The CC region contains only one form, *C. japonica* f. dissecta, while WC, EC, TW, and NA contain two forms: *C. japonica* f. dissecta and *C. japonica* f. japonica. Similarly, the SC region has three forms: *C. japonica* f. dissecta, *C. japonica* f. japonica and *C. japonica* f. pinnatisecta (Table 1; Figure 6A).

The HSTK population in the EC region has *C. japonica* f. dissecta (02) and *C. japonica* f. japonica (01 and 03). The GXLS population in the SC region has *japonica* f. japonica (32) and *C. japonica* f. pinnatisecta (33). The GDSG population includes *C. japonica* f. dissecta (50), *C. japonica* f. japonica (48) and *C. japonica* f. pinnatisecta (49). Individuals of different forms originating from the same population are still clustered together in the phylogenetic tree.

The GXHZ population has *C. japonica* f. dissecta (36), *C. japonica* f. japonica (35) and *C. japonica* f. pinnatisecta (34). The HNSY population has *C. japonica* f. dissecta (38) and *C. japonica* f. japonica (39). In the GXHZ and HNSY regions, some individuals originating from the same population did not cluster together on the phylogenetic tree, but they still corresponded to individuals from the same population in separate parts and were clustered together, regardless of the form to which they belonged (Table 1; Figure 5A).

### 3.5 PCA and bar plot of ancestry coefficient results

PCA was performed on six regions, and PC1 vs PC2 identified six groups and explained 17.45% and 11.92% of the variation, respectively (Figure 6A). The EC, CC and WC regions clustered closely together. The TW and NA regions clustered closely together. SC formed one separate group. However, the WC region still had populations embedded in the EC region, while the other populations formed separate groups. The PCA indicated that the genetic relationships among the EC region, CC region and WC region were relatively close. The relationships among these regions were similar to those in the NJ tree results (Figures 5A, B).

To understand the regional genetic structure of the six regions, we used 47,033 unlinked SNPs for STRUCTURE analysis. At *K* = 2, the NA and CC regions first diverged from the other regions and displayed two independent clusters. This indicates that both of them have relatively independent genetic backgrounds. At *K* = 3 to 5, the SC region diverged secondarily and had little genetic admixture. Although the optimal *K* determined for ADMIXTURE analysis was 7, it is noteworthy that the ADMIXTURE outcomes at K = 6 genetic clusters aligned closely with the findings from PCA and NJ analyses (Figures 6B, C). At *K* = 6, the admixtures of the individuals in the NA and TW regions and the individuals in the EC and WC regions were much closer. However, it is distinct from those of the other regions. In summary, for the relationships between these regions, all three analyses shown above demonstrated similar patterns (Figures 6B, C).

### 3.6 Genetic diversity and population structure

Based on the six regions, we detected significantly different levels of *π* between the NA region and the EC region (*p* = 0.019 < 0.05) and between the NA region and the SC region (*p* = 0.029 < 0.05) based on Kruskal‒Wallis tests. We detected significantly different levels of *H*_O_ between the NA region and the EC region (*p* = 0.012 < 0.05) and between the NA region and the SC region (*p* = 0.014 < 0.05) based on Kruskal‒Wallis tests. We detected significantly different levels of *H*_E_ between the NA region and the EC region (*p* = 0.011 < 0.05) and between the NA region and the SC region (*p* = 0.027 < 0.05) based on Kruskal‒Wallis tests. There were significantly lower levels of *F*_IS_ in the SC region (*F*_IS_ = −0.031) than in the NA region (*F*_IS_ = 0.008) (*p* < 0.05), TW region (*F*_IS_ = 0.022) (*p* < 0.05), and WC region (*F*_IS_ = −0.009) (*p* < 0.05). Furthermore, the *F*_IS_ values in the TW region (*F*_IS_ = 0.022) were significantly greater than those in the EC region (*F*_IS_ = −0.019) (*p* < 0.05) based on ANOVA.

The pairwise *F*_ST_ values between the 6 regions ranged from 0.133 to 0.722, with all of them being significant (*p* < 0.05) after sequential Bonferroni correction (Table 4).The pairwise *F*_ST_ values between the populations in the EC region ranged from −0.039 to 0.539, with approximately 4% being significant (*p* < 0.05) after sequential Bonferroni correction (Supplementary Table S1). All of the significant pairwise comparisons (2 out of 2) involved population HSTK. The average *F*_ST_ value across populations in the EC region was 0.181. The pairwise *F*_ST_ values between the populations in the WC region ranged from −0.003 to 0.909, with none being significant (*P* < 0.05) after sequential Bonferroni correction (Supplementary Table S2). The average *F*_ST_ value across populations in the WC region was 0.397. Pairwise *F*_ST_ values between the populations in the CC region ranged from 0.828 to 0.854, with none being significant (*P* < 0.05) after sequential Bonferroni correction (Supplementary Table S3). The average *F*_ST_ value across populations in the CC region was 0.842. The pairwise *F*_ST_ values between the populations in the TW region ranged from 0.258 to 0.580, with none being significant (*P* < 0.05) after sequential Bonferroni correction (Supplementary Table S4). The average *F*_ST_ value across populations in the NA region was 0.443. The pairwise *F*_ST_ values between the populations in the NA region ranged from −0.006 to 0.911, with approximately 14% being significant (*P* < 0.05) after sequential Bonferroni correction (Supplementary Table S5). Among the significant pairwise comparisons, the great majority (3 out of 4) involved population LNDD. Due to the geographical distance between the LNDD (located in Northeast China) and the Korean Peninsula and the Japanese archipelago, there was a significant difference in the *F*_ST_ values. The average *F*_ST_ value across populations in the NA region was 0.672. The pairwise *F*_ST_ values between the populations in the self-compatibility region ranged from −0.189 to 0.923, with approximately 3% being significant (*P* < 0.05) after sequential Bonferroni correction (Supplementary Table S6). Among the significant pairwise comparisons, the great majority (3 out of 5) involved populations GXHZ and HNSY. The average *F*_ST_ value across populations in the SC region was 0.375.

**TABLE 4:** Pairwise *F*_ST_ values among the 6 regions of *C. japonica*: pairwise *F*_ST_ (below diagonal) and *P*-value after 1,000 permutations (above diagonal).

	NA	EC	WC	CC	TW	SC
NA	-	0.000	0.000	0.000	0.000	0.000
EC	0.433^∗^	-	0.000	0.000	0.000	0.000
WC	0.409^∗^	0.133^∗^	-	0.000	0.000	0.000
CC	0.644^∗^	0.220^∗^	0.193^∗^	-	0.000	0.000
TW	0.220^∗^	0.396^∗^	0.382^∗^	0.722^∗^	-	0.000
SC	0.551^∗^	0.450^∗^	0.461^∗^	0.530^∗^	0.519^∗^	-

Pairwise F_ST_, values with asterisk (*) indicates a significant difference from zero following sequential Bonferroni correction (*P* < 0.05). The same applies below.

A comparison of the significant pairwise *F*_ST_ values after sequential Bonferroni correction in the EC, NA, and SC regions revealed that the *F*_ST_ of the EC region was the lowest (0.188, *P* < 0.05), that of the NA region was the highest (0.806, *P* < 0.05), and that of the SC region was 0.444 (*P* < 0.05).

### 3.7 Historical and contemporary gene flow analysis

**FIGURE 7:**
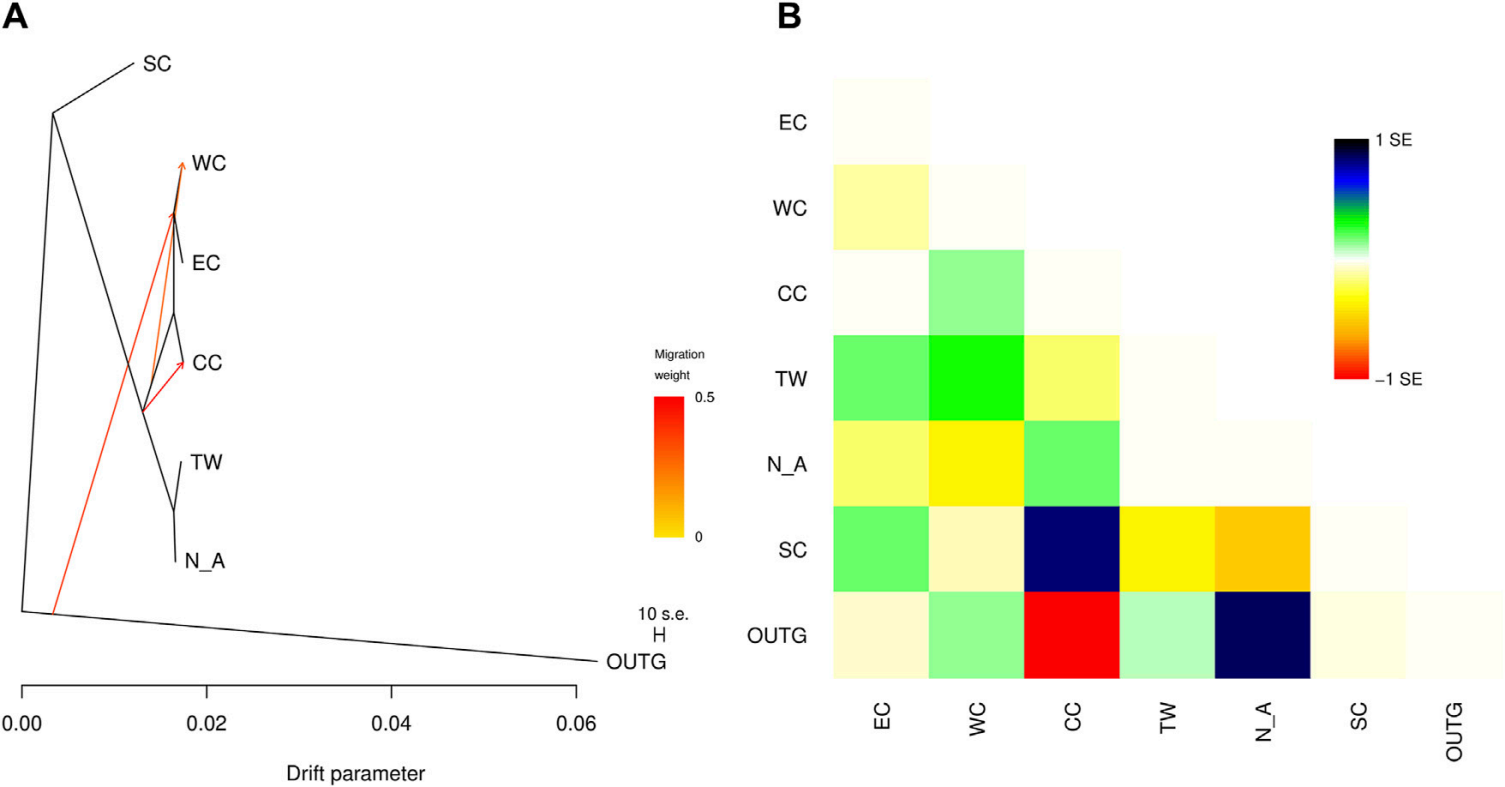
**(A)** TreeMix graph of the relationships among the sampled *C. japonica* regions. Migration events corresponding to directional gene flow are indicated by arrows. The arrow color indicates the migration weight, with darker orange indicating a stronger genetic effect on the destination region. **(B)** Scaled residuals from the fit of the model to the data.

The addition of migration events to our ML trees improved the model fit to the data (increased the model log-likelihood and decreased model residuals, but model log-likelihood increases were minimal after three migration events) (Table 5). The first migration event (Figure 7) indicated migration from their common ancestor (except the SC region) to the CC region. The second migration event suggested historical gene flow from their common ancestor to the WC and EC regions. The third migration event suggested occasional, infrequent gene flow from middle latitudes in China (including WC, CC and EC) to EC and WC (Figure 7), which implied historical gene flow in the east‒west direction. The TreeMix results indicated that WC, EC and CC were strongly differentiated from the other regions (Figure 7). These three regions (EC, CC and WC), which are located in the middle latitudes of China, were genetically distinct from the other regions but had greater similarity to the TW and NA regions than to the SC region.

**TABLE 5:** Model log-likelihood for TreeMix models with 1–12 migration events.

Migration events	Model log-likelihood
0	−1,634.94
1	−5.79
2	128.66
3	233.84
4	236.73
5	237.65
6	237.88
7	238.22
8	238.42
9	238.43
10	236.95
11	239.24
12	237.41

The proportion of populations originating from within each identified region varied from 87.9% to 97.2%, with the highest value found in the SC region and the lowest value found in the EC region (Table 6). Across the six region pairs, migrant ancestry was low (from 0.3% to 9.3%) and nonsignificant (Table 6). The posterior probability values of migration obtained from the run with the lowest estimate of Bayesian deviance suggest strong isolation for all the inferred regions.

**TABLE 6:** Matrix of inferred gene flow between genetic regions.

Gene flow	CC	EC	NA	SC	TW	WC
CC	0.907 (0.034)	0.020 (0.022)	0.018 (0.018)	0.014 (0.014)	0.022 (0.019)	0.019 (0.015)
EC	0.093 (0.032)	0.879 (0.032)	0.006 (0.006)	0.008 (0.007)	0.008 (0.009)	0.006 (0.006)
NA	0.010 (0.011)	0.012 (0.011)	0.940 (0.024)	0.014 (0.011)	0.012 (0.011)	0.012 (0.013)
SC	0.006 (0.006)	0.007 (0.007)	0.003 (0.003)	0.972 (0.010)	0.005 (0.005)	0.007 (0.006)
TW	0.026 (0.023)	0.028 (0.024)	0.021 (0.019)	0.018 (0.015)	0.882 (0.042)	0.026 (0.025)
WC	0.026 (0.025)	0.006 (0.006)	0.007 (0.007)	0.008 (0.007)	0.008 (0.009)	0.946 (0.030)

**FIGURE 8:**
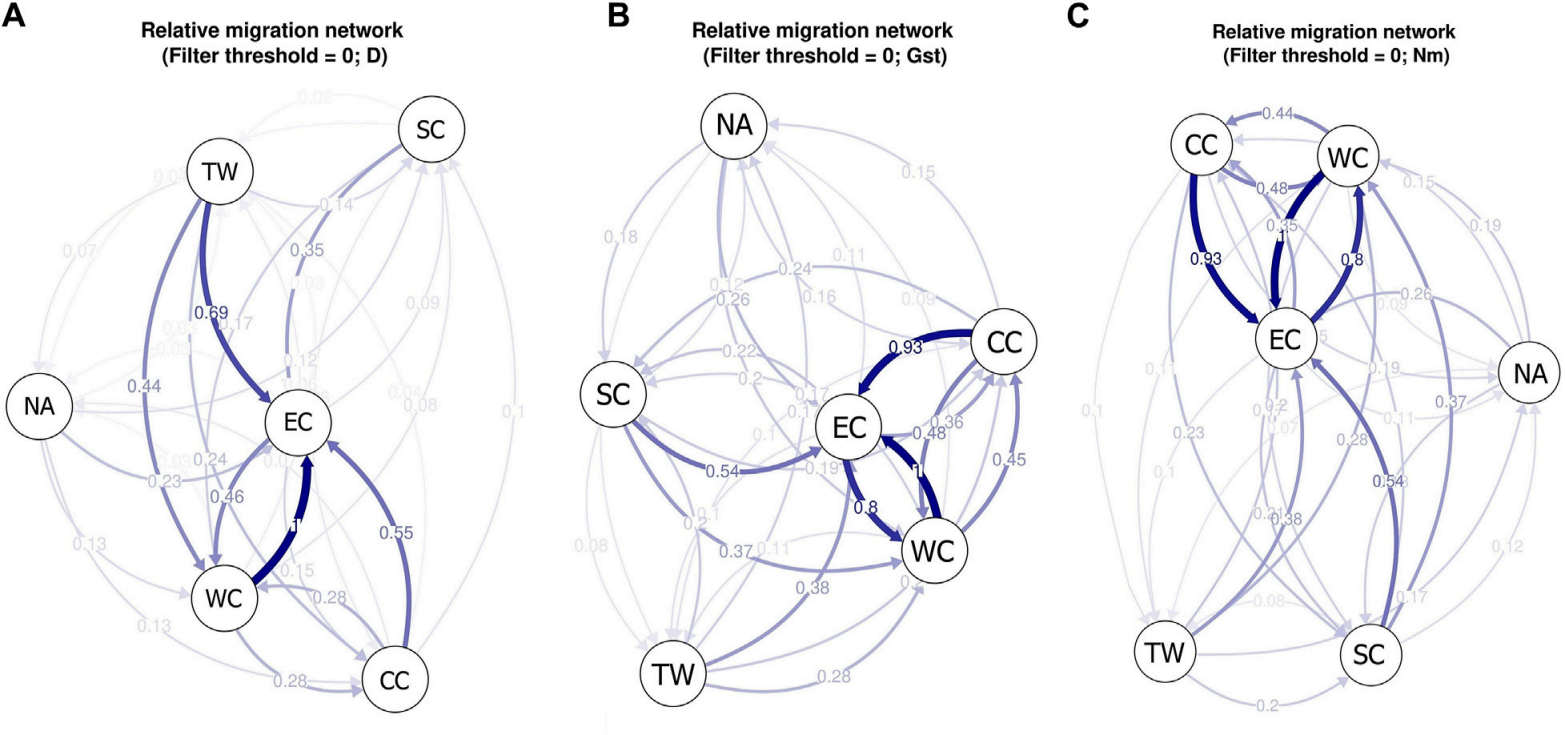
**(A)** The migration network for *C. japonica* among different geographic regions based on Jost’s D values; **(B)** The migration network for *C. japonica* among different geographic regions based on Nei’s *G*_ST_ values; **(C)** The migration network for *C. japonica* among different geographic regions based on *Nm* values. The arrows indicate the direction of gene flow. The thickness of the arrow indicates the strength of gene flow between different regions, and the number indicates the relative migration value.

Relative migration analysis of *C. japonica* using diveRsity software allowed investigation of the strength and direction of gene flow among the six regions. Based on the Jost’s D values, we observed significant asymmetric gene flow patterns in 4 out of the 6 regions. The specific result described indicates stronger migrations within the middle latitudes of East Asia (CC, SC, TW and WC regions) than within the Korean Peninsula, Japanese Archipelago and southern China (NA and SC regions) and relatively limited migration between them (Figure 8A). The EC region appears at the center of the migration network, and there is greater gene flow in this region than out of this region, which suggests that it acts as a sink for the *C. japonica* populations that emerged from imported gene flow, with limited outbound migration from this region to other regions. In addition, we observed significant asymmetric gene flow from the TW region to the EC and WC regions.

Based on Nei’s *G*_ST_ and *Nm* values, we obtained the same gene flow patterns (Figures 8B, C). The results showed that there was more significant gene flow within mainland East Asia than within the Korean Peninsula, Japanese Archipelago and Taiwan Island and relatively limited migration between them. The main reason may be the isolation between islands and the mainland during species distribution, resulting in a lack of opportunities for gene exchange between them. The SC region also showed lower imported gene flow, and the EC region appeared at the center of the migration network. The obvious difference between gene flow patterns A, B, and C was the absence of significant asymmetric gene flow from the TW region to the EC or WC region.

## 4 Discussion

The study of genetic diversity and population structure in widespread plants, such as *Quercus cerris* L. in Europe (Lados et al., 2024), *Allium macrostemon* Bunge in Japan (Probowati et al., 2023), and *Capsicum pubescens* Ruiz & Pav. in America (Palombo and Carrizo García, 2022), provides valuable insights into the factors influencing genetic differentiation. These factors include selection pressure, gene flow, and life history (Gamba and Muchhala, 2020). For long-lived species, gene flow among populations can significantly mitigate genetic erosion caused by habitat fragmentation (Fuller and Doyle, 2018). Conversely, short-lived herbs, with their rapid generational turnover, may experience a more pronounced decline in genetic diversity under similar fragmentation conditions (Young et al., 1996). Moreover, plants with abiotic-mediated pollination and seed dispersal are less susceptible to habitat fragmentation than those relying on animal-mediated mechanisms (Sato and Kudoh, 2014; Fontúrbel et al., 2015).

In the case of *Cryptotaenia japonica*, an annual or biennial herb found in damp forest areas, streams, and ditches, our AMOVA analysis revealed nearly equal genetic variation between and within populations (55.55% and 44.45%, respectively) (Table 3). This finding is consistent with the low and nonsignificant *π*, *H*_O_, *H*_E_, and *F*_IS_ values between populations and pairwise *F*_ST_ values, indicating low genetic differentiation, minimal geographic structure, and high gene flow among *C. japonica* populations across the studied regions (Tables 1, 3, and 4). Typically, outcrossing species exhibit higher genetic diversity compared to selfing species (Nybom, 2004). The negative inbreeding coefficients (*F*_IS_) observed for most populations (Table 2) suggest a lack of common inbreeding within *C. japonica* populations, which results in lower genetic differentiation but also lower genetic diversity.

Our analysis revealed no clear genetic separation based on geographic distribution for *C. japonica* populations (Figure 4). Pairwise *F*_ST_ values between populations across regions correlated weakly with geographic distances (Supplementary Tables S1, S5, S6), implying that populations within the same region share similar genetic backgrounds. This pattern is likely due to continuous and similar habitat preferences within geographic units, allowing high gene flow across relatively flat and expansive areas (Mims et al., 2016).

Previous studies have shown that water can significantly aid seed dispersal for wind-dispersed species across fragmented landscapes (Soomers et al., 2012; Yuan et al., 2022). We hypothesize that water flow may similarly facilitate the seed dispersal of *C. japonica*. Although this hypothesis remains speculative due to the lack of detailed morphological and experimental evidence, the genetic evidence presented here confirms that *C. japonica* has sufficient seed dispersal capabilities to maintain moderate-to-high levels of gene flow and population connectivity over large spatial and temporal scales.

The discontinuous distribution of *C. japonica* between mainland China and Taiwan, as well as between mainland China and Japan, can be attributed primarily to geological and climatic history. During the glacial periods of the Pleistocene epoch, lower sea levels resulted in land bridges connecting the Asian mainland to Taiwan and Japan, facilitating plant migration across these regions. Post-ice age sea level rise subsequently submerged these land bridges, isolating mainland plant populations from those on the islands (Harrison et al., 2001; Qiu et al., 2011). Climatic changes, particularly the East Asian Monsoon system, which brings wet summers and dry winters, have also significantly influenced plant distributions. The monsoon’s variable impact across the region, due to topographical differences, has led to diverse microclimates that support genetic differentiation (Qiu et al., 2011).

Gene flow events among the six regions were identified using TreeMix (Figure 7). Notably, strong gene flow was observed from the TW region to the EC region, as indicated by Jost’s D analysis (Figure 8A). The EC region emerged as a central hub for gene flow based on diveRsity gene flow analysis (Jost’s D, Nei’s *G*_ST_, and *Nm*), suggesting its role as a genetic “bridge” throughout the East Asian distribution area.

Overall, our findings highlight the complex interplay between genetic diversity, gene flow, and geographic factors in shaping the population structure of *C. japonica*. The observed high levels of gene flow and low genetic differentiation suggest that *C. japonica* has maintained connectivity across its range, despite historical and contemporary geographical barriers. This study provides a valuable reference for future research on the genetic diversity of widespread plants in Asia and underscores the importance of considering both historical and contemporary processes in understanding plant population dynamics.

## 5 Conclusion

This study provides a comprehensive analysis of the genetic diversity and population structure of *Cryptotaenia japonica* across East Asia. The findings reveal substantial genetic differentiation among populations, with significant variation in genetic diversity metrics across different regions. The lack of isolation by distance suggests that historical and ecological factors may be more influential in shaping the genetic structure of *C. japonica* populations. These insights contribute to our understanding of the genetic dynamics of widespread plant species in Asia and provide a foundation for future conservation and research efforts.

## Data Availability

The datasets presented in this study can be found in online repositories. The names of the repository/repositories and accession number(s) can be found below: https://www.ncbi.nlm.nih.gov/, PRJNA1051289.
